# Evaluation of the kinase domain of *c-KIT *in canine cutaneous mast cell tumors

**DOI:** 10.1186/1471-2407-6-85

**Published:** 2006-04-01

**Authors:** Joshua D Webster, Matti Kiupel, Vilma Yuzbasiyan-Gurkan

**Affiliations:** 1 Comparative Medicine and Integrative Biology Program, College of Veterinary Medicine, Michigan State University, East Lansing, MI, USA; 2 Department of Pathobiolgy and Diagnostic Investigation, College of Veterinary Medicine, Michigan State University, East Lansing, MI, USA; 3 Department of Small Animal Clinical Sciences, College of Veterinary Medicine, Michigan State University, East Lansing, MI, USA; 4 Department of Microbiology and Molecular Genetics, College of Veterinary Medicine, Michigan State University, East Lansing, MI, USA

## Abstract

**Background:**

Mutations in the *c-KIT *proto-oncogene have been implicated in the progression of several neoplastic diseases, including gastrointestinal stromal tumors and mastocytosis in humans, and cutaneous mast cell tumors (MCTs) in canines. Mutations in human mastocytosis patients primarily occur in *c-KIT *exon 17, which encodes a portion of its kinase domain. In contrast, deletions and internal tandem duplication (ITD) mutations are found in the juxtamembrane domain of *c-KIT *in approximately 15% of canine MCTs. In addition, ITD *c-KIT *mutations are significantly associated with aberrant KIT protein localization in canine MCTs. However, some canine MCTs have aberrant KIT localization but lack ITD *c-KIT *mutations, suggesting that other mutations or other factors may be responsible for aberrant KIT localization in these tumors.

**Methods:**

In order to characterize the prevalence of mutations in the phospho-transferase portion of *c-KIT*'s kinase domain in canine MCTs exons 16–20 of 33 canine MCTs from 33 dogs were amplified and sequenced. Additionally, in order to determine if mutations in *c-KIT *exon 17 are responsible for aberrant KIT localization in MCTs that lack juxtamembrane domain *c-KIT *mutations, *c-KIT *exon 17 was amplified and sequenced from 18 canine MCTs that showed an aberrant KIT localization pattern but did not have ITD *c-KIT *mutations.

**Results:**

No mutations or polymorphisms were identified in exons 16–20 of any of the MCTs examined.

**Conclusion:**

In conclusion, mutations in the phospho-transferase portion of *c-KIT*'s kinase domain do not play an important role in the progression of canine cutaneous MCTs, or in the aberrant localization of KIT in canine MCTs.

## Background

**Figure 1:**
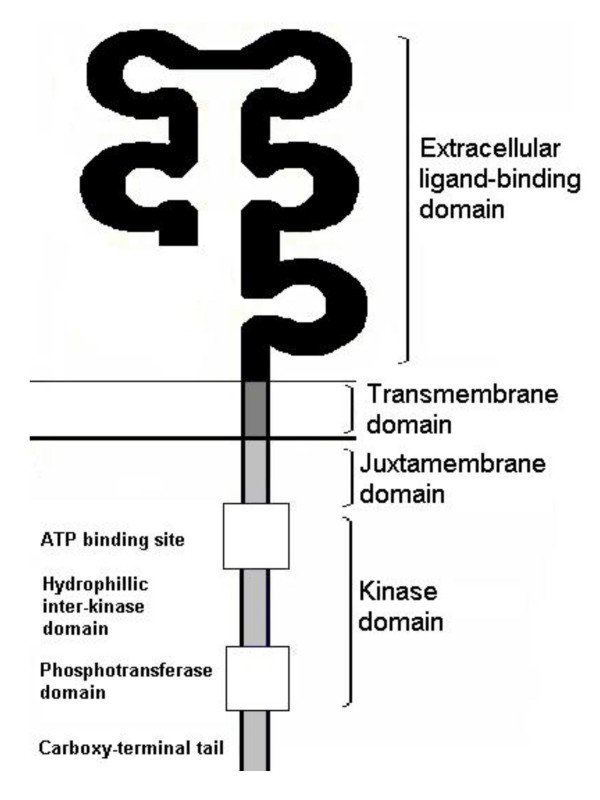
Schematic diagram of the receptor tyrosine kinase KIT.

The *c-KIT *proto-oncogene encodes the type III receptor tyrosine kinase KIT, which consists of an extracellular ligand binding domain, a transmembrane domain, a negative regulatory juxtamembrane domain and a split kinase domain [1-3] (Figure 1). In healthy humans as well as in dogs, *c-KIT *is expressed by multiple cell types including mast cells, germ cells, melanocytes, and hematopoietic precursor cells [4-8]. Notably, in mast cells KIT and its ligand stem cell factor (SCF, also known as mast cell growth factor) [9-12] have been shown to be involved in cell survival, proliferation, differentiation, chemotaxis, degranulation, and fibronectin adhesion [10,13-17]. In human patients, mutations in the *c-KIT *proto-oncogene have been implicated in the pathogenesis of multiple neoplastic diseases, including mastocytosis, germ cell tumors, and gastrointestinal stromal tumors (GISTs) [18-23]. The locations of these *c-KIT *mutations vary between the different neoplastic diseases. The vast majority of mutations characterized in human patients with mastocytosis occur at codon 816 in exon 17, which encodes a portion of the kinase domain of KIT [19,24,25]. Mutations in germ cell tumors have been found in both the juxtamembrane domain and the kinase domain of *c-KIT *[21,22], while mutations in GISTs tend to occur in exon 11 of the juxtamembrane domain of *c-KIT *[23,26,27]. Despite their variation in location both, juxtamembrane domain and kinase domains *c-KIT *mutations result in a constitutively activated KIT protein that is phosphorylated in the absence of the ligand [18,21-23].

In recent years, *c-KIT *has been implicated in the pathogenesis of canine cutaneous mast cell tumors (MCTs), which are one of the most common neoplasms in dogs [28-32]. Internal tandem duplications (ITDs), deletions, and point mutations have been identified in the juxtamembrane domain of *c-KIT *in canine cutaneous MCTs [33-35]. The reported incidence of *c-KIT *mutations has varied between different studies. In two studies that screened randomly selected cases submitted, mutations were identified in 15% of canine MCTs [35,36]. However, another study reported *c-KIT *mutations in 50% of canine MCTs that were seen in a referral oncology practice [37]. This discrepancy in the incidence of *c-KIT *mutations in canine MCTs is most likely due to the variations in case selection between these studies with the higher percentage reflecting ascertainment bias at the referral practice. Internal tandem duplication *c-KIT *mutations are the most common [35,38], and therefore the most extensively studied *c-KIT *mutation in canine MCTs. All of the ITD mutations that have been studied thus far have been found to produce a constitutively activated product, thereby implicating *c-KIT *in the progression of canine MCTs [33,34,39]. Previous work by our laboratory has shown that *c-KIT *mutations are significantly associated with higher histologic grade MCTs [35], and that MCT patients with ITDs have a significantly worse prognosis as compared to patients without ITD mutations, thereby further implicating *c-KIT *in the progression of canine MCTs [36]**.**

**Figure 2:**
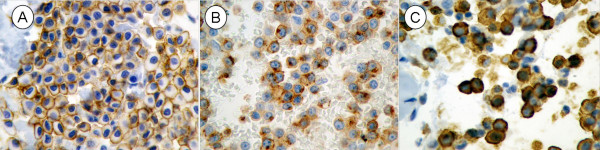
Immunohistochemical staining patterns of canine cutaneous MCTs staining with anti-KIT antibodies. A. KIT staining pattern 1: peri-membrane KIT protein localization; B. KIT staining pattern 2: focal or stippled cytoplasmic KIT protein localization; C. KIT staining pattern 3: Diffuse cytoplasmic KIT localization. Canine MCTs with KIT staining patterns 2 and 3 that lacked ITD *c-KIT *mutations were screened for mutations in *c-KIT *exon 17. B and C show MCTs with aberrant KIT localization that lack ITD *c-KIT *mutations.

In addition to *c-KIT *mutations, aberrant KIT expression and more specifically, the aberrant localization of KIT has been described in canine cutaneous MCTs [40-42]. Recently our laboratory has identified 3 patterns of KIT protein localization in canine MCTs: 1. peri-membrane KIT localization (KIT pattern 1); 2. cytoplasmic stippling to focal KIT localization (KIT pattern 2); 3. diffuse cytoplasmic KIT localization (KIT pattern 3), as shown in Figure 2. Recent studies have shown that canine MCTs that have a primarily cytoplasmic pattern of KIT protein localization (KIT patterns 2 and 3) have a significantly worse prognosis, in terms of both their disease-free interval and survival duration, as compared to MCTs that have a primarily peri-membrane pattern of KIT localization [41]. Furthermore, we have found that ITD *c-KIT *mutations in canine MCTs are significantly associated with aberrant KIT localization in neoplastic mast cells. However, a substantial number of canine MCTs have aberrant KIT localization, but do not have ITD mutations [36]. This suggests that additional factors, aside from ITD *c-KIT *mutations may be responsible for aberrant KIT localization in neoplastic mast cells.

Despite the high incidence of *c-KIT *kinase domain mutations in human patients with mastocytosis, only a total of 18 canine MCTs and 3 canine MCT cell lines have been evaluated for kinase domain mutations in *c-KIT *[33,34]. For a more detailed evaluation of possible *c-KIT *kinase domain mutations in canine MCTs, we screened exons 16–20 of the phospho-transferase portion of the kinase domain of *c-KIT *for mutations that may contribute to the progression of these tumors. Additionally, in order to determine if mutations in exon 17 of *c-KIT*, where the majority of mutations occur in human mastocytosis patients, were responsible for aberrant KIT localization, exon 17 was screened in 18 MCTs with aberrant KIT localization that lack ITD *c-KIT *mutations. However, no mutations or polymorphisms were identified in exons 16–20 of any of the canine MCTs that were examined.

## Methods

### Study population

Samples from 33 canine cutaneous MCTs from 33 dogs were obtained from archival formalin-fixed paraffin-embedded tissues that had been submitted to the Diagnostic Center for Population and Animal Health at Michigan State University. Single paraffin blocks with neoplastic tissue were selected for each tumor and each tumor was histologically graded based on the Patnaik histologic grading system [43] for canine cutaneous MCTs.

### DNA isolation from formalin-fixed paraffin embedded tissues

Tissue samples for DNA isolation were selected within the tumor boundaries, as identified by histologic evaluation. Approximately 2 mm^3 ^tissue samples were obtained from each block for DNA extraction. DNA was isolated as previously described [35,44]. In brief, 400 μl of digestion buffer (50 mM Tris, pH 8.5, 1 mM EDTA, 0.5% Tween) was added to each sample. Samples were heated to 95°C for 10 minutes followed by heating in a microwave at full power twice for 30 seconds. Samples were thoroughly vortexed between each heating step. 5 μl of 15 mg/ml proteinase K was added to each sample, and the samples were subsequently incubated overnight at 42°C. Proteinase K was inactivated at 95°C for 10 minutes. Samples were then centrifuged and 200 μl of the reaction was aliquoted for use as template in PCR.

### Amplification of c-KIT exons 16–20

**Figure 3:**
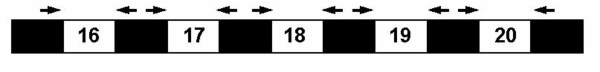
Schematic Diagram of Primer Design for Polymerase Chain Reaction. Forward and reverse primers (arrows) were designed in introns (black boxes) flanking exons 16, 17, 18, 19, and 20 (white boxes).

PCR amplification was carried out using primer pairs that flanked exons 16, 17, 18, 19, and 20 in order to sequence each exon in its entirety and the 10–20 nucleotides that flank each exon (Table 1, Figure 3). Twenty-five microliter PCR reactions were prepared with 5 μl DNA at a 1:25 dilution, 5 pmol of each primer, 0.5 units of Taq polymerase (Invitrogen, Carlsbad, CA), and final concentrations of 80 μM dNTPs, 2 mM MgCl_2_, 20 mM Tris-HCl, and 50 μl KCl. Cycling conditions for amplifying exons 16, 17, 18, and 20 were as follows: 94°C for 4 minutes; 40 cycles at 94°C for 1 minute, 60°Cfor 1 minute, and 72°C for 1 minute; 72°C for 5 minutes. Cycling conditionswere similar for exon 19, except a 54°C annealing temperature was used instead of a 60°C annealing temperature. Amplified products were visualized by agarose gel electrophoresis on a 2% agarose gel stained with ethidium bromide.

### Sequencing of c-KIT exons 16–20

Amplified products were pooled in groups of 7–10 for DNA sequencing whenever possible, as previously described [45]. This pool and sequence method has been shown to allow for the detection of minor alleles at frequencies as low as 5%. If clean sequences were not obtained from pooled samples, then samples were sequenced individually. DNA to be sequenced was separated on a 2% agarose gel, and DNA fragments were excised for DNA purification. DNA was purified using the Qiaex II gel purification kit (Qiagen, Valencia, CA) according to manufacturer's protocol. DNA sequencing was carried out using the Thermo Sequenase radiolabeled terminator cycle sequencing kit (Amersham, Piscataway, NJ), following manufacturer's instruction. Sequence reactions were separated on a 6% denaturing polyacrylamide gel, which was dried and exposed to BioMax MR scientific imaging film (Kodak, Rochester, NY) for 72 hours for visualization.

### DNA isolation from laser capture microdissected tumor samples

Eighteen MCTs with aberrant KIT localization, but no ITD *c-KIT *mutations were identified as part of a previous study [36] (figure 3. Seven micron sections of each MCT were dehydrated and stained with hematoxylin for laser capture microdissection. 2,000–4,000 neoplastic mast cells were extracted from each tumor sample using the Pixcell laser capture microdissection system with Macro LCM caps (Arcturus, Mountain View, CA). LCM caps adhered to extracted cells were incubated inverted overnight in 50 μl of DNA extraction buffer (10 mM Tris pH 8.0, 1 mM EDTA, 1% Tween) and 1.5 μl of 15 mg/ml Proteinase K at 37°C. Samples were centrifuged at 1306 × g for 5 minutes, and Proteinase K was inactivated by heating at 95°C for 8 minutes. 5 μl of DNA was used for each 25 μl reaction. PCR reactions were prepared using primers flanking *c-KIT *exon 17 as described above.

## Results

Thirty-three cutaneous MCTs from 33 dogs were included in this study. The age of these dogs ranged from 2.5–15 years with an average of 7.42 years. Twenty of the 33 dogs were female and 13 were male. The breed distribution of this study population included 10 boxers, 9 Labrador retrievers, 5 golden retrievers, 1 Boston terrier, 1 Bichon Frise and 7 mixed breed dogs. All MCTs were graded according to the Patnaik histologic grading system for canine cutaneous mast cell tumors [43]. Twelve of the 33 MCTs were histologic grade 1, 18 were grade 2, and 3 were grade 3. All 33 MCTs included in this study were previously screened for mutations in the juxtamembrane domain of *c-KIT*. No internal tandem duplications or deletions were identified in the juxtamembrane domain of *c-KIT *in any of the MCTs included in this study (data not shown).

In order to identify potential activating mutations *c-KIT *exons 16, 17, 18, 19, and 20, which encode the phospho-transferase region of the kinase domain of the KIT protein, were amplified using PCR amplification and sequenced. Exons 16 to 20 were identical to previously published cDNA sequences of the canine *c-KIT *gene [Genbank:AF448148] in all canine MCTs examined. No mutations or polymorphisms were identified in *c-KIT *exons 16 to 20 in any of the canine cutaneous MCTs screened. One single nucleotide polymorphism was identified at the 7^th ^nucleotide of intron 18 consisting of a C to A transversion.

Previous work by our laboratory has identified a correlation between ITD *c-KIT *mutations and aberrant KIT localization in canine cutaneous MCTs. However, we have also found that a subset of canine cutaneous MCTs have aberrant KIT localization without having ITD *c-KIT *mutations [36]. In human mastocytosis patients, mutations of *c-KIT *are commonly seen in exon 17 [19,24,25]. In order to test the hypothesis that mutations in *c-KIT *exon 17 are responsible for aberrant KIT localization in canine MCTs that lack ITD *c-KIT *mutations, exon 17 was amplified and sequenced from 18 such cases. Exon 17 was identical to previously published *c-KIT *sequences [Genbank:AF448148] and no mutations or polymorphisms were identified in exon 17 in any of the canine MCTs evaluated.

## Discussion

This study did not identify any mutations of the phospho-transferase domain of *c-KIT *in 33 canine cutaneous MCTs examined. These findings are also supported by reports from London et al. in which 11 MCTs were examined for kinase domain mutations [33]. Therefore, it is highly unlikely that the phospho-transferase domain of *c-KIT *plays a role in the progression of canine MCTs. Furthermore, mutations in exon 17 of the *c-KIT *proto-oncogene, where mutations are most commonly seen in human patients with mastocytosis [19,24,25], do not contribute to the aberrant localization of KIT in canine cutaneous MCTs.

**Figure 4:**
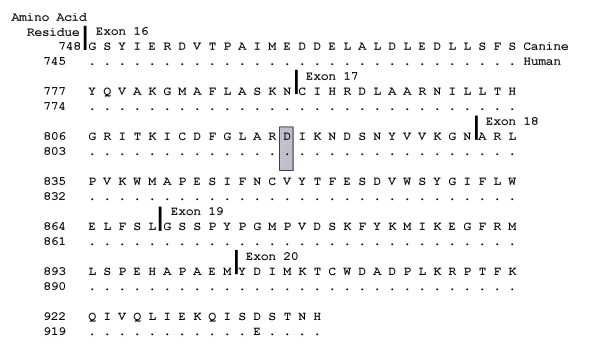
Amino acid alignment of the kinase domain (exons 16–20) of canine and human KIT. Canine and human amino acid alignments demonstrate a 99.5% identity between the phospho-transferase portion of the kinase domain of these species. Codon 816, which is commonly mutated in human mastocytosis patients (D816V; shaded residue) is also conserved in canine KIT.

The kinase domain of *c-KIT *is highly conserved between humans and canines. In a comparison of the human [Genbank: NM_000222] and canine [Genbank: AF448148] amino acid sequences corresponding to exons 16–20, there is only a single difference in which a glutamate in the human is replaced with an aspartate residue in exon 20 of the dog, giving greater than 99% identity between these species (figure 4). The high degree of conservation between the human and the dog suggests that a change in a single amino acid of the kinase domain could alter the function of the KIT protein, potentially producing a constitutively activated protein, as is seen in human mastocytosis patients. However, unlike human mastocytosis patients, kinase domain *c-KIT *mutations do not appear to play a role in the progression of canine MCTs.

Internal tandem duplications and deletions in the juxtamembrane domain of *c-KIT *have been identified in approximately 15% of canine MCTs [36]. The internal tandem duplication mutations are the most frequent and best characterized *c-KIT *mutations in canine MCTs [35,38]. All of the characterized ITD mutations result in a constitutively activated KIT product, which is characterized by the constitutive phosphorylation of the receptor in the absence of ligand binding [33,34,39]. Considering the role of *c-KIT *in mast cell survival and proliferation [10,17]and the association between ITD *c-KIT *mutations and decreased disease-free interval and survival duration of dogs [36], *c-KIT *appears to play a key role in the progression of canine MCTs [36]. Therefore, *c-KIT *may also represent a potential therapeutic target for canine cutaneous MCTs [35-37,39,41,46].

Juxtamembrane domain *c-KIT *mutations have only been found in approximately 15% of all canine MCTs [35,36] and 50% of high grade MCTs [37]. We hypothesized that phospho-transferase domain *c-KIT *mutations may play a role in the progression of canine cutaneous MCTs that lack ITD *c-KIT *mutations. The goal of this study was to investigate the presence of any activating mutations in the phospho-transferase domain of the *c-KIT *proto-oncogene. The results of this study suggest that *c-KIT *mutations of the phospho-transferase domain do not play a significant role in the progression of canine MCTs. Since some small molecule kinase inhibitors have shown a greater efficacy in tumors with mutations in the target receptor tyrosine kinase (e.g. KIT) [39,47,48], MCTs lacking *c-KIT *mutations may not be as good of candidates for the treatment with these drugs. Therefore, other genetic or epigenetic changes that play a role in the progression of canine MCTs need to be identified in order to develop targeted treatment strategies in those MCTs that lack ITD *c-KIT *mutations. However, additional domains of the *c-KIT *proto-oncogene still need to be evaluated for the presence of activating mutations in canine MCTs. One area of particular interest is, the extracellular ligand-binding domain where mutations have been identified in exon 8 of human familial mastocytosis and GIST patients [49] and exon 9 of human GIST patients [48,50,51].

Additional work from our laboratory has shown a correlation between ITD *c-KIT *mutations and the aberrant localization of KIT in canine MCTs [36]. These results suggest that ITD *c-KIT *mutations may lead to the aberrant cytoplasmic localization of KIT in at least a subset of canine MCTs. Despite this correlation between ITD mutations and aberrant KIT localization, we have identified some MCTs that have aberrant KIT localization, but do not have ITD *c-KIT *mutations [36]. We hypothesized that point mutations in exon 17 of the *c-KIT *proto-oncogene, similar to those seen in human patients with mastocytosis, are responsible for the aberrant KIT localization in MCTs without ITD *c-KIT *mutations. However, in this study we did not find any mutations in exon 17 of the *c-KIT *proto-oncogene in 18 canine cutaneous MCTs with aberrant KIT localization. These results suggest that other changes to *c-KIT*, such as mutations in other *c-KIT *domains, changes in transcriptional regulation or alternative splicing, or other cellular changes, such as changes in golgi processing or intracellular trafficking may be responsible for aberrant KIT localization and may therefore play a role in the progression of canine cutaneous MCTs.

## Conclusion

In this study no kinase domain *c-KIT *mutations were found in any canine cutaneous MCT examined and therefore these mutations do not appear to play a significant role in the progression of this disease. In accordance with this data, exon 17 *c-KIT *mutations do not appear to be responsible for aberrant KIT localization in canine MCTs with aberrant KIT localization that lack ITD *c-KIT *mutations. Due to the relatively low incidence of juxtamembrane domain *c-KIT *mutations, and the lack of kinase domain mutations in canine cutaneous MCTs, further studies are necessary to identify additional factors and genes that lead to the progression of canine cutaneous MCTs. Although targeting constitutively activated KIT protein in canine MCTs offers great promise for the treatment of canine MCTs [39,46], such a therapeutic approach may not be justified for all MCTs. A significant number of MCTs seem to have no alterations in the *c-KIT *proto-oncogene, and targeted inhibition of this gene will therefore have little or no effect on these tumors. Therefore, additional studies need to be performed in order to determine other factors that may be involved in the progression of canine MCTs without ITD *c-KIT *mutations in order to identify potential targets in those tumors.

## Competing interests

The author(s) declare that they have no competing interests.

## Authors' contributions

JDW carried out the PCR amplification and sequencing of this study, with technical assistance from Daniel Zemke, Elizabeth Kruszewski, and Angela Sosin, and drafted the manuscript.

MK participated in the design of the study and helped draft the manuscript.

VYG participated in the design of the study, coordinated the study and helped draft the manuscript.

All authors red and approved the final manuscript.

## Pre-publication history

The pre-publication history for this paper can be accessed here:



## Figures and Tables

**Table 1:** Primers used for PCR amplification of *c-KIT *exons 16–20 and size of expected PCR products for each primer pair.

**Exon**	**Primer Number**	**Direction**	**Sequence**	**Product Size (bp)**
16	1202	Forward	CTT TGA GGC TTA ATT GCT AAG AA	256
	1203	Reverse	ACT ATG AAC TCT AAA ATG CGC CA	
17	1204	Forward	ATA GCA GCA TTC TCG TGT TG	261
	1205	Reverse	AAC TAA AAT CCT TCA CTG GAC TG	
18	1206	Forward	AAC ATT GCC GGA TCT GTT GT	189
	1207	Reverse	AGA TGC TCT CGC CCA ACC A	
19	1208	Forward	GGG TCC TGC TTG CTT ATT	188
	1209	Reverse	AGC ATG ATC TCA AGG GAA	
20	1210	Forward	AGG CTA AGG GCG TTG AGG	189
	1211	Reverse	GCA GGG AGG TTC TAC GGC T	
